# Reliability Prediction of Ontology-Based Service Compositions Using Petri Net and Time Series Models

**DOI:** 10.1155/2014/760202

**Published:** 2014-02-06

**Authors:** Jia Li, Yunni Xia, Xin Luo

**Affiliations:** ^1^Chongqing Technology and Business Institute, Chongqing 400052, China; ^2^Software Theory and Technology Chongqing Key Lab, Chongqing University, Chongqing 40030, China

## Abstract

OWL-S, one of the most important Semantic Web service ontologies proposed to date, provides a core ontological framework and guidelines for describing the properties and capabilities of their web services in an unambiguous, computer interpretable form. Predicting the reliability of composite service processes specified in OWL-S allows service users to decide whether the process meets the quantitative quality requirement. In this study, we consider the runtime quality of services to be fluctuating and introduce a dynamic framework to predict the runtime reliability of services specified in OWL-S, employing the Non-Markovian stochastic Petri net (NMSPN) and the time series model. The framework includes the following steps: obtaining the historical response times series of individual service components; fitting these series with a autoregressive-moving-average-model (ARMA for short) and predicting the future firing rates of service components; mapping the OWL-S process into a NMSPN model; employing the predicted firing rates as the model input of NMSPN and calculating the normal completion probability as the reliability estimate. In the case study, a comparison between the static model and our approach based on experimental data is presented and it is shown that our approach achieves higher prediction accuracy.

## 1. Introduction

Web Services are interfaces that describe a collection of operations that are network-accessible through standardized protocols. Web services in the Semantic Web are described through ontologies, which represent formally the service features by using a semantic mark-up language that follows a logical paradigm. One of the most used ontologies to specify Semantic Web service compositions is OWL-S (Ontology Web Language for Services) [1], which uses the OWL language capabilities to represent the service and enables agents to make inferences about it. OWL-S is a computer-interpretable semantic mark-up language, where ontology-based descriptions of service functionality and of interaction service behaviour coexist.

Recently, various studies have discussed how to effectively model and predict the reliability of service compositions based on the aggregations of the reliabilities of its constituent activities. These studies share a common idea that they all try to fit historical reliability data of activities into assumed distributions (deterministic, exponential, geometrical, or general distributions) and employ these obtained distributions as the static model input into the static stochastic models (continuous Markovian model, discrete Markovian model, or PERT (Program Evaluation and Review Technique, [2]) mode, etc.) to obtain the predicted reliability. A comprehensive investigation and discussion of these studies are given in the Section.

Our aim in this paper is therefore the development of a dynamic reliability prediction approach for service compositions, with special attention to control flow modelling of OWL-S processes and the dynamics of service quality in runtime environment. This research employs the OWL-S service processes as the example. The main innovation includes a feature-completed translation from OWL-S to Non-Markovian stochastic Petri net (NMSPN for short) and an analytical reliability method using the predicted quality parameters (using the ARMA model) as model input. In the case study, we obtain the experimental reliability data of actual service ontologies and conduct a comparative study between our approach and the static models. The comparison suggests that our dynamic prediction model produces less errors and achieves higher prediction accuracy.

## 2. Related Studies

During the last decade, the QoS of web services and service compositions has received a lot of attention in the research community. Reference [3] presents the seminal work on this topic, where the three most important quality parameters, namely, completion time, cost, and dependability, to describe the QoS of composite services at the task level are proposed. This study also proposes a stochastic workflow reduction model (SWR) approach to graphically capture the control flow and predict the QoS. To simplify the problem, this approach assumes execution times and the normal-completion-rates of service tasks to be deterministically distributed rather than random. These deterministic values can be directly derived from the Service-Level-Agreement (SLA).

In this case, a composite service is equivalent to a static PERT network with deterministic edges. The service completion time can be easily calculated as the longest execution path of the PERT network. Similar works can be found in [4–7], where deterministic execution times of tasks are assumed.

It is easy to see that the aforementioned assumption of deterministic task execution times and normal completion rates of service tasks is unrealistic for real-world scenarios. Recent studies therefore assume randomly distributed variables as the model input instead. To simplify the solution, exponential or geometric distributions of tasks are assumed in [8–11] and our studies of [12, 13] to derive a Markovian state-space and achieve closed-form expressions of QoS results. These studies all rely on the assumption of Markov property. In probability theory, a stochastic process has the Markov property if the conditional probability distribution of future states of the process, given the present state and all past states, depends only upon the present state and not on any past states; that is, it is conditionally independent of the past states (the path of the process) given the present state (also termed the memory-less property). However, the Markov property is often an unrealistic assumption and rarely satisfied by real scenarios. In reality, tasks may have arbitrary distributions (in some extreme cases, for instance, some safety-critical systems tasks are required to take deterministic delay for the purpose of obtaining fully predictable system behaviours and bounded turn-around-time).

Recently, a step forward is taken by assuming generally distributed task execution times and reliabilities. For example, the work in [14] assumes that tasks in composite services follow discrete-general-distributions, where distributional functions of task execution time maps outcome to values of a countable set. Although the assumption of general distribution provides a more refined characterization of real distributions of task execution times and reliabilities than that of deterministic variables and exponential variables, it still has limitations. In deriving the probability-distributional-functions (PDF) of QoS of service activities, these studies simply employ the historical empirical distribution as the actual PDF and assume that such PDF is capable of characterizing even the future changes of QoS. In doing so, the cumulative ratio or density of QoS metric equalling a certain value is considered to be the future rate of equalling such value, while ignoring the trend and seasonality by which the historical QoS values increase or decrease. For example, consider the execution times of tasks. The empirical PDF may suggest a normal distribution. However, the empirical PDF may fail in capturing the fact that the density of execution time being low decreases, probably caused by the deteriorating connectivity of network. By contrast, a time series analysis may reveal the up-going trend and suggest a tailed distribution. In this case, only a time series-based prediction of future execution times guarantees the correctness and accuracy.

## 3. The ARMA Model

Autoregressive/moving average (ARMA) [14] models have been successfully used to model and predict QoS of network, ATM, manufacturing system, and so forth. We now show that they can be used to model the QoS of service activities in composite service flows as well. Intuitively an ARMA(1, 1) model captures the correlated nature of a time varying distribution through a combination of an autoregressive (AR) component and a moving average (MA) component. The parameter (1, 1) describes the number of terms in the AR and MA components, respectively. For a stationary time series *X*_*t*_, we define an ARMA(*p*, *q*) process as

(1) $$\begin{array}{c} \phi \left(B\right)X_{t}=\theta \left(B\right)Z_{t}, \end{array}$$

where *ϕ*(*B*) is the autoregressive polynomial of degree *p* and *θ*(*B*) is the moving average polynomial of order *q*,

(2) $$\begin{array}{c} \phi \left(B\right)=1-\phi_{1}B-\cdots -\phi_{p}B^{p},\\ \phi \left(B\right)=1+\theta_{1}B+\cdots +\theta_{q}B^{q}, \end{array}$$

and *B* is the back-shift operator defined as

(3) $$\begin{array}{c} B^{i}X_{t}=X_{t-1}, \end{array}$$

where *i* = 0, ±1, ±2,….

The innovations *Z*_*t*_ are assumed to be independent identically distributed random variables with zero mean and variance *σ* ^2^. If *θ*(*Z*) = 1 we have a pure autoregressive (AR) process, while if *ϕ*(*z*) = 1 we have a pure moving average process (MA).

To predict the future value of an established ARMA(*p*, *q*) series, Box-Jenkins method is used. Box-Jenkins ARMA model requires series to be stationary. Intuitively a time series is stationary if the statistical properties such as the mean and the variance are not time dependent. Generally if the values of the time series fluctuate about a constant mean value without a trend, then the time series is stationary. Box-Jenkins method involves four iterative steps that must be followed as follows: series stationarity, model identification, optimization and selection, and model diagnostic checking.

The details of these iterative steps can be found in [14] and are therefore omitted in this paper. The prediction program can be easily implemented using the standard procedures for the Box-Jenkins ARMA model analysis in Matlab 7.0.

## 4. Model Translation of OWL-S

In this section, we introduce the translation rules for OWL-S. Because the syntax of OWL-S is too vast, we restrict the translation into a subset (OWL-S elements such as input-binding, output-binding, data manipulation, boolean condition evaluation, preconditions, and results are abstracted away and omitted), which describes the control flows of the activity executions and message exchanges. This subset is mainly specified by the *Service grounding *and* Service model* specifications. It captures the control flow evolution of the OWL-S workflow, the compositional patterns by which the processes are organized, and the invocation of external services (from abstract processes to the concrete services specified by the WSDL documents).

Firstly, translation rule for atomic process is presented. The atomic process in OWL-S is a description of a service that expects one (possibly complex) message and returns one (possibly complex) message in response. It is invoked through the 〈*perform*〉 construct. It corresponds to an action that a service can perform in a single interaction, and it can be executed in a single step by sending and receiving appropriate messages. The main operation of The atomic process is to contact and invoke the partner services through the grounding mechanism. The grounding is a mapping from the abstract specifications to actual executable services (specified by WSDL documents).

**Figure 1:**
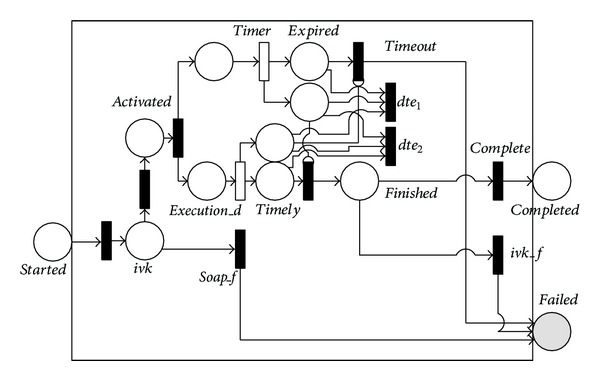
NMSPN model of the atomic process.

According to the discussion above, an atomic process in OWL-S can be translated into the NMSPN model given in Figure 1. In Figure 1, the *started* and *completed* places indicate the initial and completed states of the process, respectively. The *execution_d* timed transition denotes the duration needed for the invoked service to complete execution. *Timer* denotes the timeout threshold. Because the external services are invoked through SOAP messages and the SOAP connections are subject to failure (caused by message loss, for instance), the *soap_f* transition is used to capture the SOAP failure and it directly marks *failed* (in gray) to indicate the unsuccessful invocation. If no SOAP failure occurs and the service execution duration exceeds the timeout threshold, a timeout event is triggered and the *failed* place is marked. Otherwise, the *timeout* transition is prevented and the *timely* place is marked. In this case, the execution of the invoked external service can either be faulty (by firing the *ivk_f* transition and marking the *failed* place) or successful (by firing the *complete* transition and marking the *completed* place). Note that the *dte*_1_ and *dte*_2_ (*dte* stands for dead-token-elimination) transitions ensure that no dead token exists when the atomic process normally completes or fails.

In the following, we present translation rules for composite processes. All composite processes are composed of atomic processes. The 〈*composedOf*〉 property in OWL-S describes the control flow and the data flow of subprocesses within a composite process, yielding constraints on the ordering and conditional execution of these subprocesses. The composite processes can be implemented by the 〈*sequence*〉, 〈*split*〉, 〈*split*-*join*〉, 〈*choice*〉, 〈*any*-*order*〉, 〈*if*-*then*-*else*〉, 〈*repeat*-*while*〉, and 〈*repeat*-*until*〉 patterns.

**Figure 2:**
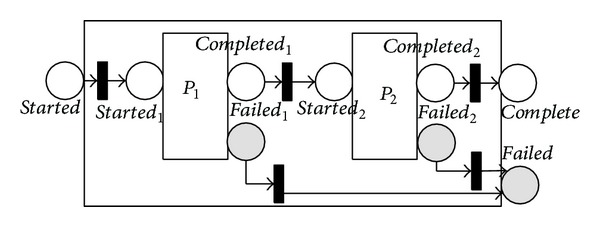
NMSPN model of the sequence process.

We start with the 〈*sequence*〉 process. In this process, a list of control constructs is executed sequentially. The equivalent NMSPN model of this process is given in Figure 2. For simplicity, we assume that there exist only two subprocesses, namely, *P*_1_ and *P*_2_. The failure mode of 〈*sequence*〉 is simply implemented by propagating the inner failures of *P*_1,2_ to the level of 〈*sequence*〉 itself through the immediate transitions from *failed*_1,2_ to *failed*.

**Figure 3:**
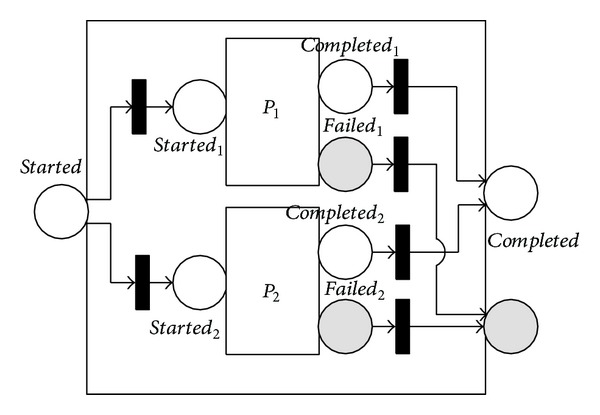
NMSPN model of the choice process.

The 〈*choice*〉 process stipulates that a single one from a given group of subprocesses (specified by the 〈*components*〉 property) is executed. As shown in Figure 3, the choice construct includes two selective branches, *P*_1_ and *P*_2_. It is organized by an XOR selective construct. Selecting and completing either branch would allow the 〈*choice*〉 process to finish. The failure mode of 〈*choice*〉 is implemented in a similar way to that of the 〈*sequence*〉 process.

**Figure 4:**
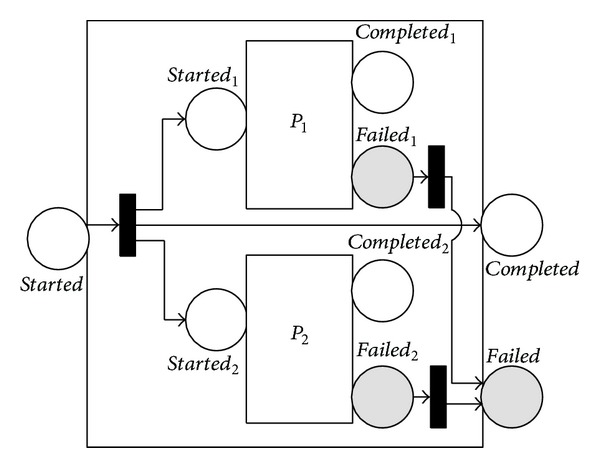
NMSPN model of the split process.

The 〈*split*〉 process stipulates that branches are executed in parallel. It completes as soon as all of its branches have been scheduled for execution and does not wait for the completion of those branches. The translation of the 〈*split*〉 process is given in Figure 4.

**Figure 5:**
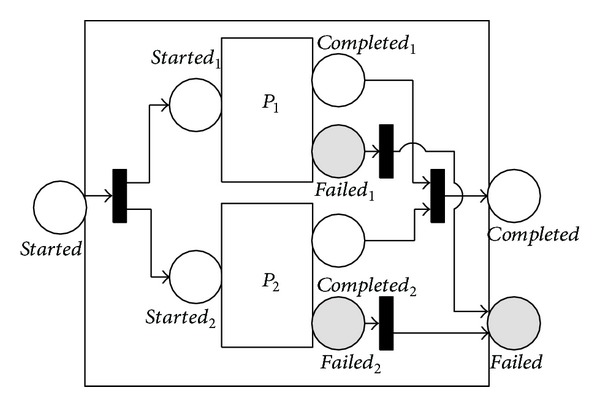
NMSPN model of the split-join process.

The 〈*split*-*join*〉 process also supports concurrent execution but is intrinsically different from the 〈*split*〉 process. It consists of the concurrent execution of a bunch of processes, following a barrier synchronization style. That is, it completes when all of its subprocesses have completed. The equivalent NMSPN model of the 〈*split* + *join*〉 process is given in Figure 5.

**Figure 6:**
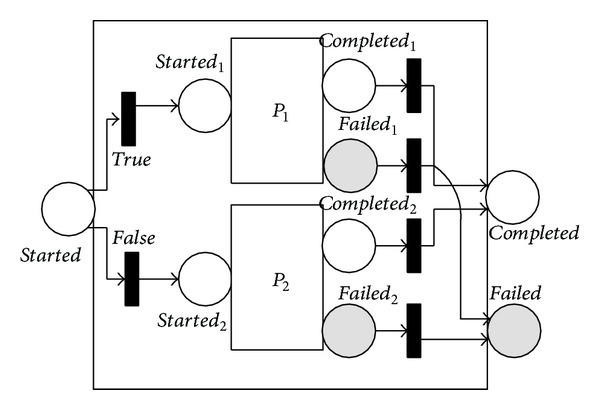
NMSPN model of the if-then-else process.

The 〈*if*-*then*-*else*〉 process is a control construct associated with a Boolean decision. If the condition is satisfied, the true branch (i.e., the 〈*then*〉 branch) is selected and executed, otherwise the false branch (i.e., the 〈*else*〉 branch). The 〈*if*-*then*-*else*〉 process is accomplished when its selected branch is completed. The equivalent NMSPN model is given in Figure 6, where the *true/false* immediate transitions denote the true/false evaluation of the Boolean condition.

**Figure 7:**
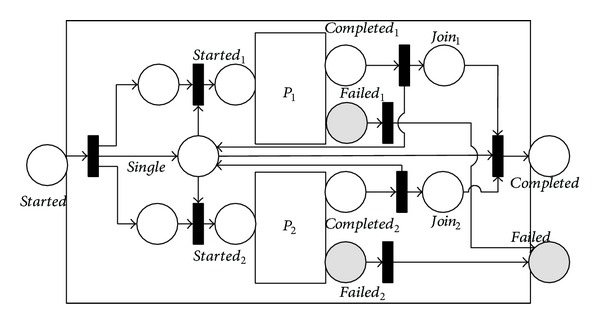
NMSPN model of the any-order process.

The 〈*any*-*order*〉 process allows the subprocesses to be executed in some unspecified order but not concurrently. Execution and completion of all branches are required. As shown in Figure 7, the execution of branches in an 〈*any*-*order*〉 process cannot overlap and all branches must be executed before the 〈*any*-*order*〉 process completes. The *single* place and the bidirectional arcs from *single* guarantee that *P*_1_ and *P*_2_ are not executed concurrently.

**Figure 8:**
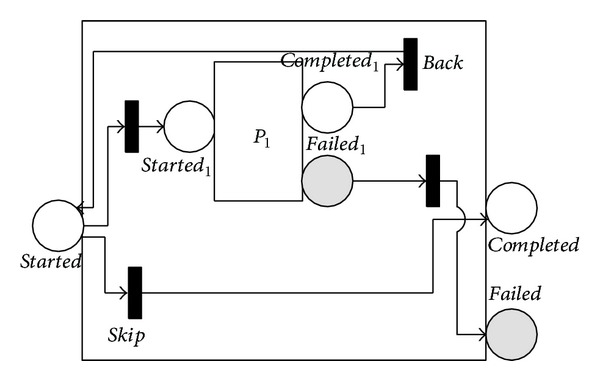
NMSPN model of the repeat-while process.

**Figure 9:**
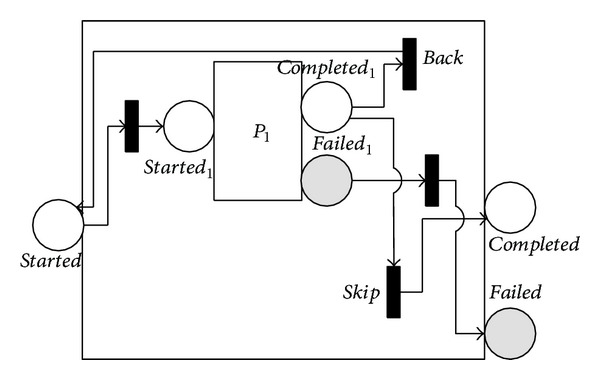
NMSPN model of the repeat-until process.

Both the 〈*repeat*-*while*〉 and 〈*repeat*-*until*〉 processes support iterative execution. They keep iterating until a condition becomes false or true. 〈*repeat*-*while*〉 tests for the loop condition loop if the condition is true and otherwise execute the nested process. 〈*repeat*-*until*〉 executes the nested process, tests for the condition, exits if it is true, and otherwise loops. Thus, 〈*repeat*-*while*〉 may never execute its nested process, whereas 〈*repeat*-*until*〉 always executes the nested process at least once. Figures 8 and 9 show the NMSPN models of the two processes. In these figures, the *back* immediate transition leads the control flow back to the beginning, and the *skip* immediate transition leads the control flow out.

## 5. A Case Study

**Figure 10:**
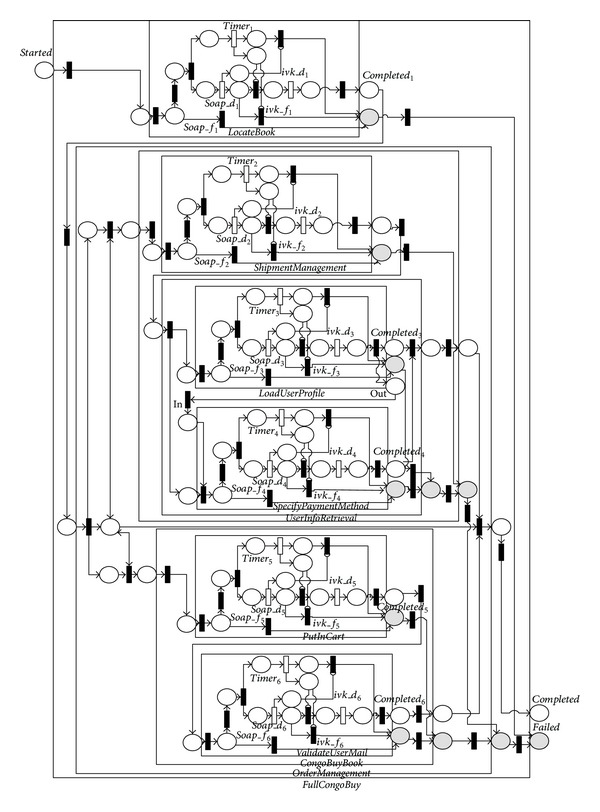
NMSPN model of the any-order process.

In this section, we conduct a case study to illustrate the effectiveness of the translation introduced above. The case study is based on the frequently used *CongoProcess* sample given in [15]. *The FullCongoBuy* process is the uppermost composite process of the sample. It is organized by a 〈*sequence*〉 process and composed of an atomic process, *LocateBook*, and a composite process, *OrderManagement*. The *OrderManagement* process implements an 〈*any*-*order*〉 process and includes two composite processes, namely, *CongoBuyBook* and *UserInfoRetrieval*. The *UserInfoRetrieval* process implements sequential process and includes two atomic processes, namely, *LoadUserProfile* and *ValidateUserEmail*. The *CongoBuyBook* process also implements sequential processes and includes a composite process, *BuySequence*. The *BuySequence* process implements a sequential process and includes an atomic process, *PutInCart*, and a composite process, *SignInAndSpecify*. The *SignInAndSpecify* process implements a 〈*split*-*join*〉 process and includes two atomic processes, namely, *SpecifyPaymentMethod* and *ShipmentManagement*. Based on the translation rules given in the previous section, the sample can be translated to the NMSPN model given in Figure 10.

## 6. Predicting the Firing Rates of Timed Transitions

As discussed earlier, timed transitions correspond to two kinds of activities in the atomic processes, namely, the timeout activities and activities of executing invoked services. The former always have invariable delays (prescribed by system settings) and the latter always have nondeterministic delays. Instead of the static evaluation of model inputs, we dynamically fit the historical data of these nondeterministic delays into a time series model and predict the future firing rates using the ARMA method. The prediction is implemented as follows.

For any nondeterministic timed transition, its empirical firing rate at time *t* can be obtained as

(4) $$\begin{array}{c} r\left(t\right)=\frac{N}{\sum_{1\leq i\leq N,i\in N}dL\left(t+\left(i-1\right)\times inv^{\prime }\right)}, \end{array}$$

where *dL*(*t*) denotes measured delay at time *t*, *N* denotes the number of test samples with the time window, and *inv*′ denotes the time interval of samples. This equation suggests that the firing rate can be calculated as the reciprocal of the mean of the *N* delay samples within the time window of [*t*, *t* + (*N* − 1)*inv*′].

Thus, the firing rates measured at different times can be described as a sequence of data with equal time intervals:

(5) $$\begin{array}{c} R\left(t\right)=\left\{r\left(t-\left(p-1\right)\times inv\right),r\left(t-\left(p-2\right)\times inv\right)\cdots r\left(t\right)\right\}, \end{array}$$

where *r*(*t*) denotes the firing rate at time *t*, *p* is the volume of the time series, and *inv* is the interval of the time series.

Employing *R* ^*t*^ as the model input, we can use the Box-Jenkins method to predict the future firing rate at time *t* + *intv*. Since the Box-Jenkins method is well established and commonly used, its detailed process is omitted.

## 7. Reliability Prediction

In this section, we introduce the analytical methods to predict reliability of OWL-S process. This method takes the predicted fire rates (based on ARMA methods discussed earlier) and the NMSPN representations as model inputs. We use process-normal-completion-probability (PNCP) as the metric of reliability. From the NMSPN view, PCNP denotes the probability that the *completed* place of the outer-most process is marked and no *failed* place is marked.

To predict PNCP, we first have to predict reliabilities of individual atomic processes. Let *P*{*failed*} denote the probability that place *failed* in Figure 1 is marked, the reliability of the atomic process, *RAP*, can therefore be calculated as

(6) $$\begin{array}{c} RAP=1-P\left\{failed\right\}. \end{array}$$

According to earlier discussions, the *failed* place in Figure 1 can be marked for three reasons, namely, the SOAP error (by firing immediate transition *soap_f*), the triggering of timeout (by firing *timeout*), and faults of invoked external services (by firing *ivk_f*). *P*{*failed*} is therefore calculated as

(7) P{failed}=pre(soap_f)+(1−pe(soap_f))×PTE +(1−pe(soap_f))×(1−PTE)×pe(ivk_f),

where *pe*(*soap*_*f*) and *pe*(*ivk*_*f*) denote the probabilities of firing *soap*_*f* and *ivk_f*, respectively. *PTE* denotes the probability of triggering timeout. *PTE* is calculated as

(8) $$\begin{array}{c} PTE=P\left\{to<delay_{i}\right\}, \end{array}$$

where *to* denotes the timeout threshold and *delay*_*i*_ denotes the execution duration of the timed transition *soap*_*d*_*i*_.  *P*{*to* < *soap*_*d*_*i*_} can be approximately calculated as

(9) $$\begin{array}{c} P\left\{to<delay_{i}\right\}\approx e^{-rate_{i}\times to}, \end{array}$$

where *rate*_*i*_ is the predicted future firing rate using the ARMA model.

Prediction of composite processes is easier. For example, the reliability of 〈*sequence*〉 process in Figure 2, *RSE*, is

(10) $$\begin{array}{c} RSE=RP_{1}\times RP_{2}, \end{array}$$

where *RP*_1,2_ denote reliabilities of processes *P*_1,2_. Note that this equation also applies to the 〈*split*〉, 〈*split* + *join*〉, and 〈*any* *order*〉 processes.

The reliability of 〈*if*-*then*-*else*〉 process, *RIF*, is decided by reliabilities of its two branches and the probability for the associated boolean condition to be true. *RIF* is therefore calculated as the weighted (by the firing probabilities of *true* and *false* in Figure 6) reliabilities of its two branches:

(11) $$\begin{array}{c} RIF=pe\left(true\right)\times PR_{1}+pe\left(false\right)\times PR_{2}. \end{array}$$

The prediction of 〈*repeat*-*while*〉 process needs more effort. Its reliability is decided by the reliability of the embedded processes and the number of repetitive executions. Letting *NRE* denote the number of repetitive cycles to achieve completion (by marking the *completed* place in Figure 8), we have that the reliability for 〈*repeat*-*while*〉, *RRW*, is calculated as the total probability of

(12) $$\begin{array}{c} RAW=\overset{}{\underset{0\leq i\leq \infty }{\sum }}{RP}_{1}^{i}\times P\left\{NRE=i\right\}, \end{array}$$

where *P*{*NRE* = *i*} denotes the probability that *NRE* equals *i*. Since the theoretical distribution of *NRE* is difficult to evaluate (some studies suggest that the probability of skipping repetition when every iteration ends can be approximately seen stable and the number of repetitive cycles therefore follows the geometrical distribution and employ the expectation of geometrical distribution in evaluating the QoS of repetitive constructs, e.g., [4, 16, 17]), we can approximately calculate *P*{*NRE* = *i*} as the occurrence rate of *NRE* equalling *i* based on the experimental test or historical logs. That is

(13) $$\begin{array}{c} P\left\{NRE=i\right\}\approx \frac{frq\left(NRE\;equaling\;i\right)}{NT}, \end{array}$$

where *frq*(*NRE* *equalling* *i*) denotes the frequency of the event of *NRE* equalling *i* and *NT* denotes the number of experimental trials.

As discussed earlier, the 〈*repeat*-*until*〉 process also supports the repetitive execution. However, it differs from the 〈*repeat*-*until*〉 process because it tests the loop condition after the first round of executing *P*_1_. That is to say that the total round of executing *P*_1_ is the number of repetitive execution of *P*_1_, *NRE*, plus the inevitable first round:

(14) $$\begin{array}{c} RRW=\overset{}{\underset{0\leq i\leq \infty }{\sum }}{RP}_{1}^{i+1}\times P\left\{NRE=i\right\}. \end{array}$$

## 8. Experiments and Validation

To prove the feasibility and accuracy of our model, we execute the OWL-S sample given in Section 3 on the OWL-S API tool [18] and conduct a confidence-interval-analysis. The OWL-S API tool is a Java API for programmatic access to read, execute, and write OWL-S documents. The API provides an execution engine that can invoke atomic processes with WSDL groundings and composite processes that uses OWL-S control constructs. It also provides log files on the starting time and ending time of each service component and the state changes of processes. The six atomic processes are implemented by services specified by the WSDL grounding document at http://www.daml.org/services/daml-s/0.7/CongoGrounding.daml. The timeout threshold for all of the service invocation is $500 ms$.

**Figure 11:**
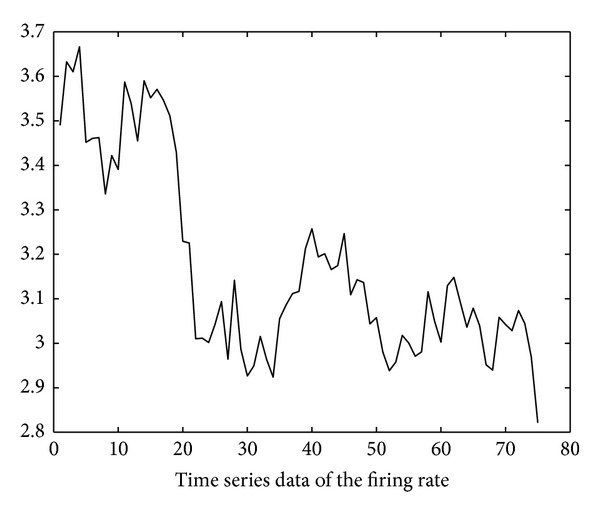
Firing rate series of LocateBook.

**Figure 12:**
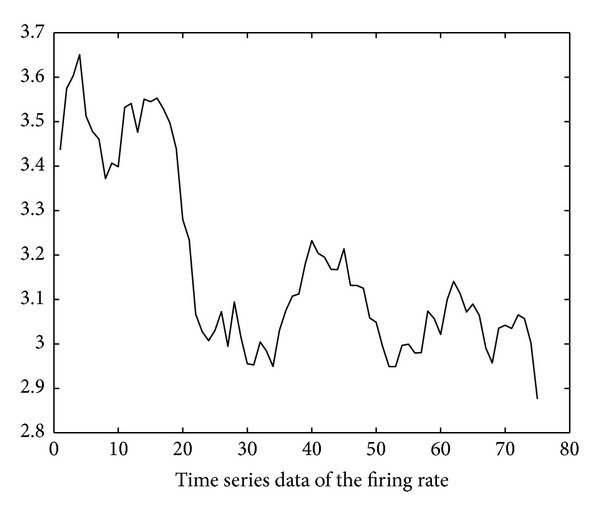
Firing rate series of ShipmentManagement.

**Figure 13:**
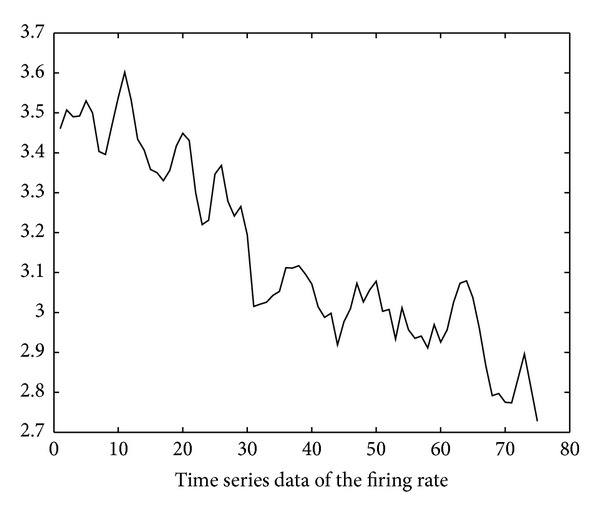
Firing rate series of LoadUserProfile.

**Figure 14:**
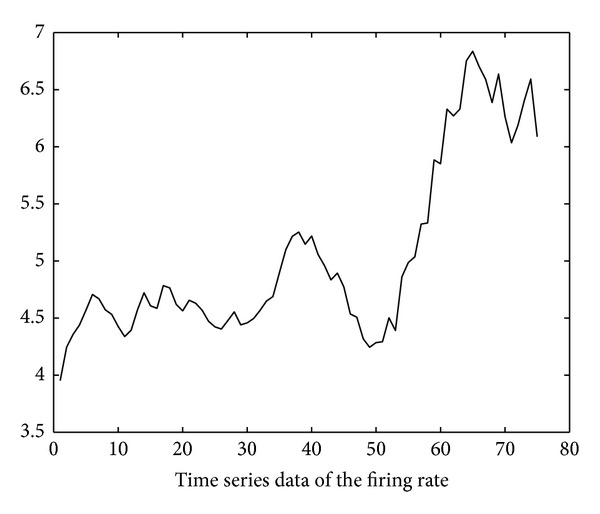
Firing rate series of SpeficyPaymentMethod.

**Figure 15:**
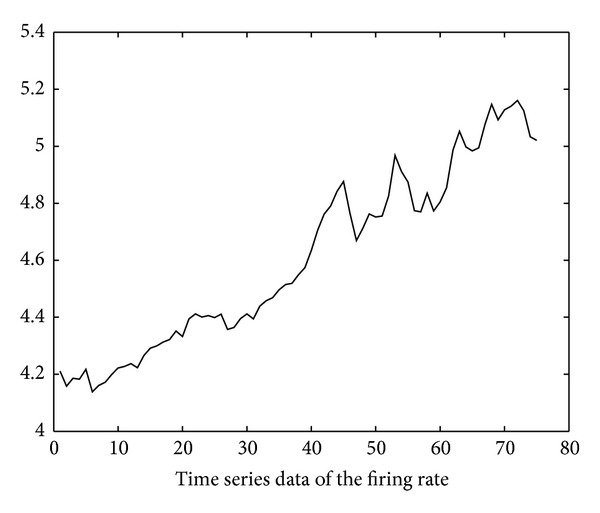
Firing rate series of PutInCart.

**Figure 16:**
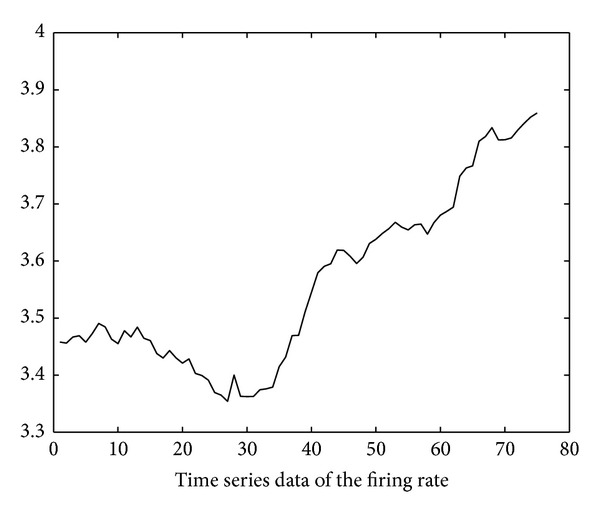
Firing rate series of ValidateUserMail.

Using the soapUI [19] test tool, we also obtain the execution delay data of the six invoked services through repetitive SOAP invocation tests. The test started at time 10:00 on November 10, 2012. The invocation interval is 250 ms (i.e., *inv*′ in (4) is 250 ms). Based on these data, the time series of firing rates of the six invoked services can be derived using (4)–(14) and is illustrated in Figures 11, 12, 13, 14, 15, and 16. Note that the interval of the series is 500 ms (i.e., *inv* in (5) is 500 ms).

We also obtain the SOAP failure connection rates of the six atomic processes as 0.0042, 0.0019, 0.0068, 0.0075, 0.0054, and 0.0048. That is to say, firing probabilities of *pe*(*soap*_*f*_1−6_) in (7) are 0.0042, 0.0019, 0.0068, 0.0075, 0.0054, and 0.0048. Moreover, we obtain the service failure rates of the six invoked operations as 0.0035, 0.0014, 0.0032, 0.0078, 0.0053, and 0.0025. That is to say, firing probabilities of *pe*(*ivk*_*f*_1−6_) in (7) are 0.0035, 0.0014, 0.0032, 0.0078, 0.0053, and 0.0025.

**Figure 17:**
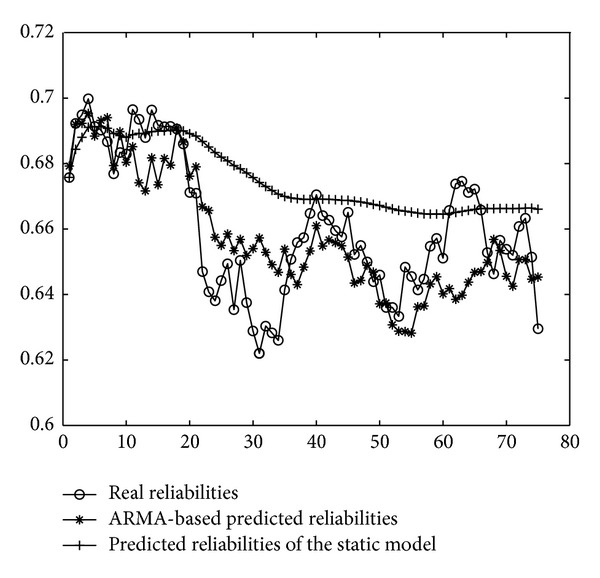
The comparison of the predicted PNCP and the actual reliabilities.

Using the ARMA as the prediction model and the firing rates series shown in Figures 11–16 as model input, we can predict the future firing rates of the *soap*_*d*_1−6_ transitions. Employing the predicted firing rates as the input of the NMSPN model, we can predict the future PNCP of the OWL-S process. The predicted PNCP results are compared with the actual probabilities of normal completion in Figure 17.

Figure 17 also shows the results of predicted reliability based on the static method mentioned in Section 2. It is easy to see that our approach achieves a better curve-fitting and a better characterization of reliability fluctuation.

## 9. Conclusions

In this paper, we present a comprehensive dependability prediction model for OWL-S processes. We first introduce a set of translation rules to map the process-level elements of OWL-S into the Non-Markovian stochastic Petri net (NMSPN). Based on the NMSPN representations, we employ the ARMA-based time series method to predict the future firing rates of the execution delays of invoked external services. Using these firing rates as model input, we introduce an analytical method to calculate the process-normal-completion-probability as the predicted future reliability. In the case study of real-world OWL-S ontologies, we show that our approach achieves higher prediction accuracy than the static evaluation model.

Based on the current research, we are also considering further studies as follows:

1. developing tools for automatic translation of OWL-S processes and reliability/dependability computation. We are currently cooperating with the Center of National Software Engineering of Peking University to integrate our prediction models into their automatic formal translation tools. It is hoped that a new tool for automatic Petri-net-translation will be realized;
2. introducing methods to predict other metrics such as performance, mobility, reputation, maintainability, and reusability.
3. Developing selection and scheduling algorithms. Based on our research, the reliability of a given composite OWL-S service process can be calculated. However, sometimes we are more interested in knowing, for a given process definition, how to choose from many functionally identical but qualitatively different services and schedule services to achieve the best reliability. We are therefore developing selection and scheduling algorithms based on the NMSPN-based reliability model.
